# Education did not interact with major depression on performance of memory tests in acute southern Brazilian in patients

**DOI:** 10.1590/S1980-57642008DN10100005

**Published:** 2007

**Authors:** Analuiza Camozzato Fleck, Marcelo Pio de Almeida, Vera Delgado, Marcia Lorena Fagundes Chaves

**Affiliations:** 1 MD, PhD Medical Sciences Post-Graduation Course and Neurology Service,Hospital de Clinicas de Porto Alegre,Universidade Federal do Rio Grande do Sul, Porto Alegre, Brazil.; 2 MD, PhD, Psychiatry Service Hospital de Clinicas de Porto Alegre,Universidade Federal do Rio Grande do Sul, Porto Alegre, Brazil.

**Keywords:** depression, neuropsychological tests, memory, cognition, education, Brazil, depressão, testes neuropsicológicos, memória, cognição, educação, Brasil

## Abstract

The relationship of cognitive function to depression in older adults has become a topic of extensive clinical interest and research.

**Objective:**

To analyze association between cognitive/memory performance,Major Depression, and education in 206 inpatients from the Psychiatry and Internal Medicine Departments.

**Methods:**

Patients were evaluated by the Mini Mental State Examination, a battery of memory tests, and the Montgomery-Åsberg Depression Rating Scale. Depression patients comprised 45 severe and 42 mild/moderate, according to the Montgomery-Asberg scale. The effect of psychoactive drugs was recorded (30% drug-free). Education was measured in years. Cognitive/memory tests assessed five domains: general mental functioning, attention, sustained attention/working memory, learning memory (verbal), and remote memory. An index for memory impairment was created (positivity: 50% of tests below cutoff).

**Results:**

The chief effect on worse performance was Major Depression for the domains (age and education adjusted) of attention, learning, remote memory, and general functioning. For the domain “sustained attention and working memory”, only severely depressed patients differed from the medical controls (p=.008). Education showed an independent effect on test performances. No interaction between depression and educational status was observed.We also observed an independent effect of psychoactive drugs on some cognitive/memory domains. Logistic Regression showed Major Depression as the main risk for cognitive impairment.

**Conclusions:**

These data demonstrated association of Major Depression with impaired cognitive performance independent of educational attainment or psychiatric medications.

## Abstract (pt)

A relação entre função cognitiva com depressão em adultos mais velhos tem se tornado um tópico de grande interesse clínico e investigativo.

**Objetivo:**

Analisar a associação entre desempenho cognitivo/ memória, depressão maior e educação em 206 pacientes das unidades de Psiquiatria e Medicina Interna.

**Métodos:**

Pacientes foram avaliados pelo Mini Exame do Estado Mental, uma bateria de testes de memória e escala de depressão de Montgomery-Åsberg. Pacientes deprimidos eram 45 graves e 42 leve/moderados de acordo com a escala Montgomery-Asberg. O efeito de drogas psicoativas foi registrado (30% não usavam medicações). Educação foi registrada em anos completos de escola. Testes cognitivos/memória avaliaram cinco domínios: função mental geral, atenção, atenção sustentada/memória operante, aprendizado (verbal), e memória remota. Um índice para comprometimento de memória foi criado (positividade: 50% dos testes abaixo do ponto de corte).

**Resultados:**

O principal efeito para pior desempenho nos domínio de atenção, aprendizado, memória remota e função geral foi depressão maior (ajustado idade e educação). Para o domínio atenção sustentada e memória operante apenas os pacientes gravemente deprimidos diferiram dos controles clínicos (p=0,008). Educação mostrou efeito independente sobre o desempenho nos testes. Nenhum efeito de interação entre depressão e status educacional foi observado. Também observamos efeito independente das drogas psicoactivas sobre os mesmos domínios cognitivos/memória. A Regressão Logística mostrou depressão maior como o maior fator de risco para comprometimento cognitivo.

**Conclusões:**

Estes dados demonstraram associação de depressão maior com desempenho cognitivo alterado independente de nível educacional ou medicações psiquiátricas.

The association of cognitive dysfunction with depression in older adults has been a topic of extensive attention.

The observations that:

1) depression would be the cause of dementia^1-3^;
2) cognitive deficits may occur in both structural and functional mental disorders^4-7^;
3) affective states interfere with encoding and retrieval of acquired items^2,8-11^; and,
4) cognitive changes are among the main goals of psychotherapy for depressed patients^12-15^ have been addressed in the literature.

A revival of interest in testing patients with depression on a wide range of neuropsychological tasks has occurred in the last decade, provoking a growing awareness that, akin to other psychiatric and neurologic disorders, mood disorders may be associated with a distinctive pattern of cognitive impairment^16^. However, these impairments are seldom quantified. An attempt to establish a profile of neuropsychological deficits for clinically depressed patients was carried out by means of a metaanalysis published in 1997^17^. This meta-analysis analyzed investigations published between 1975 and 1996 and took into consideration several methodological criteria. The findings suggested a diffuse impairment of brain function. A more recent review targeted the role of the dorsal and ventral aspects of the prefrontal cortex and interactions between affect, motivation and cognitive function in depression^16^.

Among demographic variables, measures of impact of various cultural aspects are complex, especially in subsets of different cultures within the same population. Education can be considered as an element of culture^18^ and includes literacy and schooling.

Formal education is the most significant element in culture, and both have significant effects on cognition^19^. Education has an important influence on cognitive test performance, whereby groups with higher levels of education perform better on most neuropsychological tests^19-25^. An implication of this influence is the need for careful evaluation of any psychometric or psychological test or scale in subsets of a population.

We hypothesized that educational attainment would be an interaction factor for depression to significantly affect cognition. The main goal of the present study was the analysis of performance in cognitive tests in currently depressed patients, comparing this with medical inpatients, and to evaluate the impact of educational attainment, age and gender.

## Methods

The study was carried out using a cross-sectional design.We selected patients admitted to the Psychiatric Unit, during the first 48 hours after admission, who met DSM-IV criteria for a current major depressive episode (major depressive disorder). At the same time, patients admitted to the Internal Medicine Unit were evaluated for the study (comprising the comparison group). Inclusion criteria for these patients were presence of acute illnesses without global systemic disturbances and being highly functional before hospital admission, whereas exclusion criteria were presence of any psychiatric or neurologic disease and use of psychoactive drugs. The WHO Self-Report Questionnaire – SRQ^26,27^ screened mental disorders among these patients. Eight positive questions was the cutoff for mental disorder among women, and seven among men^27^. Controls also did not meet criteria for Major Depression (DSM IV).

Psychoactive drugs for Major Depression patients administered during the last month were classified into four categories: none, antidepressants (mostly selective serotonin reuptake inhibitors), and antidepressants with other psychiatric drug (benzodiazepine, lithium, and neuroleptics). Use of benzodiazepines within 6 hours before interview was also an exclusion criteria. Severely depressed patients were distributed according to categories of drug use as 34% (N=15) none, 38% (N=17) antidepressants, and 29% (N=13) antidepressants with other psychiatric drug. The mild/moderate patients were 35% (N=15) none, 36% (N=15) antidepressants, and 26% (N=11) antidepressants with other psychiatric medication. There was no significant statistical difference between the two groups (chi-square=0.347; p=0.963).

All participants were assessed by the Montgomery-Äsberg Depression Rating Scale^28,29^. Educational attainment was given in years. The neuropsychological battery included tests that assessed five general domains: general mental functioning, attention, sustained attention and working memory, learning memory (verbal), and remote memory. General mental functioning was measured with the Mini-Mental State^30,31^. Attention was assessed with the word span^31,32^, while sustained attention and working memory with the both digit span and immediate recall of the Wechsler’s Logical memory test^32^. Learning was measured by the delayed retrieval of the word list and Logical memory^32^. Remote memory was assessed with the Major Public Events, Famous Faces and Autobiographic data tests^33,34^.

We developed an index for the evaluation of cognitive impairment through an epidemiological strategy that assesses tests in parallel to enhance diagnostic power (sensitivity and specificity)^31^. For the index, we applied cutoffs to tests, and analyzed a combination of 50% of positive results as the outcome.

The sample consisted of 206 inpatients, 87 from the Psychiatry Unit and 119 from the Medical Unit. This sample size was sufficient to detect a difference of 20% (with an error of 5%) in attention test performance (OR=3 and N=65 in each group) between depressed and healthy comparison subjects^35^.

Table 1 presents demographic characteristics of sample. The depression group included 65 women and 22 men, with age range from 19 to 76 years (mean ± standard deviation, 43.13±11.63) and mean education 8.12± 10.82 years (1 to 19). The Montgomery-Åsberg depression rating scale presented mean ± standard deviation, 30.24±11.85for the forty-two patients with mild/ moderate symptoms (<30) and 45 with severe (≥30) symptoms. The medical group consisted of 71 women and 48 men, mean age 45.83±9.^50^ (20 to 78), years of education 7.05±3.58(1 to 16), Montgomery-Åsberg 5.08±4.58 (mild symptoms), and the Self-Report Questionnaire 3.16±1.59.

**Table 1:** Demographic data from major depression and medical inpatients.

	Depression		
Variables	Severe (N=45)	Mild/moderate (N=42)	Medical inpatients (N=119)
Age (mean±SD)*	45.18±11.21^a^	40.93±11.80^b^	45.83±9.50^c^
Education (mean±SD)**	8.24±3.64	8.00±4.042	7.05±3.58
Gender - male (%)***	14 (31%)^a^	8 (19%)^b^	48 (40%)^c^
M-A scale (mean±SD)****	39.36±7.66^a^	20.48±6.53^b^	5.08±4.58^c^
SRQ (mean±SD)	-	-	3.16±1.59

SD, standard deviation; %, percentage;

* one-way ANOVA, F=3.52; p=.051-b≠c (p=0.025);

** one-way ANOVA, F=2.186; p=.115;

*** chi-square=6.48; p=0.039 (a,c≠b);

**** p=0.0001 (a,b≠c and a≠b); M-A scale,Montgomery-Åsberg scale; SRQ, self-report questionnaire.

The study was approved by the Ethics Committee for Medical Research at Hospital de Clinicas de Porto Alegre, and was conducted according to the principles established in the Helsinki declaration. Patients signed an informed consent after the nature of all procedures had been fully explained, and patient confidentiality was maintained.

### Statistical analysis

Groups were first compared on demographic and clinical variables by using analyses of variance (one-way ANOVA), chi-square analyses, and Student t tests.

The analyses of neuropsychological test data were carried out in a hierarchical fashion. First, all test scores were converted to z scores, corrected according to standards from external normative study groups (N=87, age range= 19–76). Domain scores were then calculated by averaging the z scores of the primary measure for each test within each domain (general mental functioning, attention, sustained attention and working memory, learning memory [verbal], and remote memory). Domain scores were input into a multivariate analysis of variance (MANOVA) comparing three groups. Educational attainment was recoded to a two-level factor ("7 [incomplete first grade education] and >7 years [at least complete first grade education]) for the MANOVA interaction analysis. Age entered the equation as a covariant. The main effect of gender was tested but since no significant impact alone, or as interaction was observed it is not presented.

Logistic Regression was used to determine main multivariate association with learning/memory impairment. For Logistic Regression, the following parameters are presented: B (regression coefficient) S.E. (an estimate of the standard deviation for the error terms in regression), Wald, Odds Ratio (OR) and the 95% Confidence Interval (CI) with lower and upper limits.

## Results

### Assessment of age effect

Table 2, shows mean±SD of tests classified into cognitive/ memory domains. The comparisons between groups were adjusted for age. Age correlated with Mini-Mental (B= –0.043; p=0.001), word span (B= –0.013; p=0.002), delayed recall of the word list (B= –0.027; p=0.002) and Logical memory (B= –0.029; p=0.005), famous faces (B= –0.081; p=0.0001), autobiographical data (B= –0.020; p=0.001), and Montgomery-Asberg depression rating scale (B=0.162; p=0.002) (MANOVA covariance: withinsubject effect for the whole sample).

**Table 2:** Mean±standard error of test scores of studied groups and frequency of cognitive deficit - multivariate procedures of MANOVA (adjusted for age and education).

	Depression		Medical inpatients		
Tests	Severe (N=45)	Mild/moderate (N=42)	(N=119)	F	p value *
**General mental functioning**					
Mini Mental	24.89±0.36^a^	26.08±0.38^b^	27.47±0.23^c^	17.51	0.0001
**Attention**					
Word span	4.77±0.19^a^	4.87±0.21^b^	5.45±0.13^c^	4.85	0.001
**Sustained attention and working memory**					
Digit span	5.98±0.29	6.50±0.31	6.16±0.19	0.81	0.501
Logical memory I	4.13±0.31^a^	4.48±0.33^b^	5.51±0.20^c^	7.24	0.001
**Learning memory (verbal)**					
Word list D	1.53±0.26^a^	2.04±0.28^b^	2.77±0.17^c^	7.72	0.001
Logical memory D	3.66±0.33^a^	3.81±0.35	4.51±0.21^c^	2.49	0.075
**Remote memory**					
Autobiographical	7.71±0.18^a^	8.54±0.19^b^	9.46±0.12^c^	31.55	0.0001
Major public events	3.99±0.60^a^	5.26±0.64	5.54±0.39^c^	2.32	0.216
Famous faces	13.90±0.60^a^	14.71±0.64	16.01±0.39^c^	4.25	0.008

Mini-Mental, a≠b (p=0.003), b≠c (p=0.016) and a≠c (p=0.0001);Word span, a≠b (p=0.005) and b≠c (p=0.003); Logical memory I, a≠c (p=0.0001) and b≠c (p=0.012);Word list delayed, a≠c (p=0.0001) and b≠c (p=0.029); Logical memory delayed, a≠c (p=0.042); Autobiographical, a≠b (p=0.006), b≠c (p=0.001) and a≠c (p=0.0001);Major events, a≠c (p=0.038); Famous faces, a≠c (p=0.005);

* adjusted for multiple comparisons.

### Assessment of depression effect

Diagnosis of depression presented an effect upon the Mini Mental (p=0.0001),Word span (p=0.001), the delayed recall of the Word list (p=0.001), Logical memory immediate recall (p=0.001), Autobiographical data (p= 0.0001), and famous faces (p=0.008) (Table 2).

### Assessment of education effect

The effect of educational attainment, as a two class factor, showed significant differences for Mini Mental (p=0.0001), Autobiographical data (p=0.0001), Logical memory immediate (p=0.001) and delayed recall (p= 0.001), Digit span (p=0.006),Word span (p=0.023), Famous Faces (p=0.003), and Major Public Events (p= 0.049) (Table 2).

**Figure 2:**
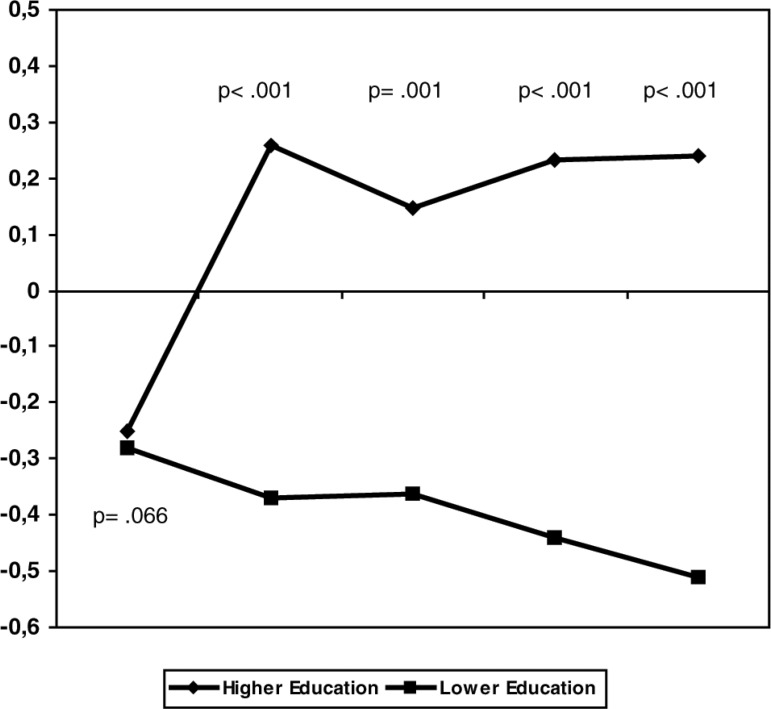
Domain z-scores according to educational attainment (>7 and = 7 years).

Education showed an independent effect on tests performances (Table 4 and Figure 2). No interaction between depression and educational status was observed.

**Table 4:** Multivariate effect upon domain mean z scores of factors under investigation (diagnostic status, educational attainment and interaction between diagnosis and education).

Factors and domains (mean z score)	F	p value
**Education**		
Attention	3.415	0.066
Sustained	17.510	<0.001
Learning	11.983	0.001
Remote	23.212	<0.001
General mental functioning	29.805	<0.001
**Diagnosis**		
Attention	4.848	0.009
Sustained	3.586	0.030
Learning	7.080	0.001
Remote	11.455	<0.001
General mental functioning	17.508	<0.001
Diagnosis * Education		
Attention	2.33	1.00
Sustained	0.79	0.457
Learning	1.04	0.354
Remote	2.04	0.132
General mental functioning	0.29	0.734

### Use of anti-depressives and antipsychotics and interaction of variables

An additional analysis was carried out with depressed patients alone, severe and mild/moderate, psychoactive drugs and education as independent variables. Dependent variables were cognitive/memory tests and age as covariant. The effect of education was the same as observed above, as was correlation of age with tests. The scores on delayed recall of the word list and on Mini Mental were higher among drug-free depressed patients, than those who were taking antidepressants with other psychiatric medications (p=0.001 and p=0.002, respectively). The patients who were taking antidepressants alone also showed higher test scores than those an antidepressants with other psychiatric medications (p=0.005 and p=0.038, respectively). Drug-free patients showed higher scores on immediate and delayed recall of logical memory than patients taking antidepressants (p=0.001) or antidepressants with other psychiatric medications (p=0.027 and p=0.001, respectively) (Table 3). Effect of severity of depression was similar to that presented in Table 2.

**Table 3:** Mean±standard error of statistically different test scores among depression patients classified by drug use - and z scores for all cognitive/memory domains multivariate procedures of MANOVA (adjusted for severity of depression, education and age).

			Antidepressants +
Tests	None (N=26)	Antidepressants (N=32)	other psychiatric drug (N=24)
**General mental functioning**			
Mini-Mental	26.87±0.48^a^	26.12±0.45^b^	24.62±0.52^c^
**Learning memory (verbal)**			
Word list D	2.85±0.30^a^	2.31±0.30^b^	1.08±0.31^c^
Logical memory D	5.84±0.39^a^	3.08±0.37^b^	3.44±0.42^c^
**Sustained attention and working memory**			
Logical memory I	5.84±0.38^a^	3.12±0.36^b^	4.57±0.42^c^
**z Score (mean SD) for each cognitive domain**			
Attention	-0.79±0.23	-0.62±0.22	-0.30±0.29
Sustained attention	0.55±0.19^a^	-0.57±0.19^b^	-0.11±0.25^c^
Learning	0.71±0.19^a^	-0.21±0.18^b^	-0.63±0.23^c^
Remote	-0.48±0.19	-0.43±0.18	-0.44±0.24
General mental functioning	0.14±0.20^a^	-0.11±0.19^b^	-0.84±0.25^c^

Mini-Mental, a>c (p=0.002) and b>c (p=0.038); Word list D, a>c (p=0.001) and b>c (p=0.005); Logical memory D, a>b (p=0.001) and a>c (p=0.001); Logical memory I, a>b (p=0.001) and a>c (p=0.027); P values adjusted for multiple comparisons; Sustained, a>b (p=0.0001) and a>c (p=0.040); Learning, a>b (p=0.001) and a>c (p=0.0001); General mental, a>c (p=0.003); P values adjusted for multiple comparisons.

**Figure 1:**
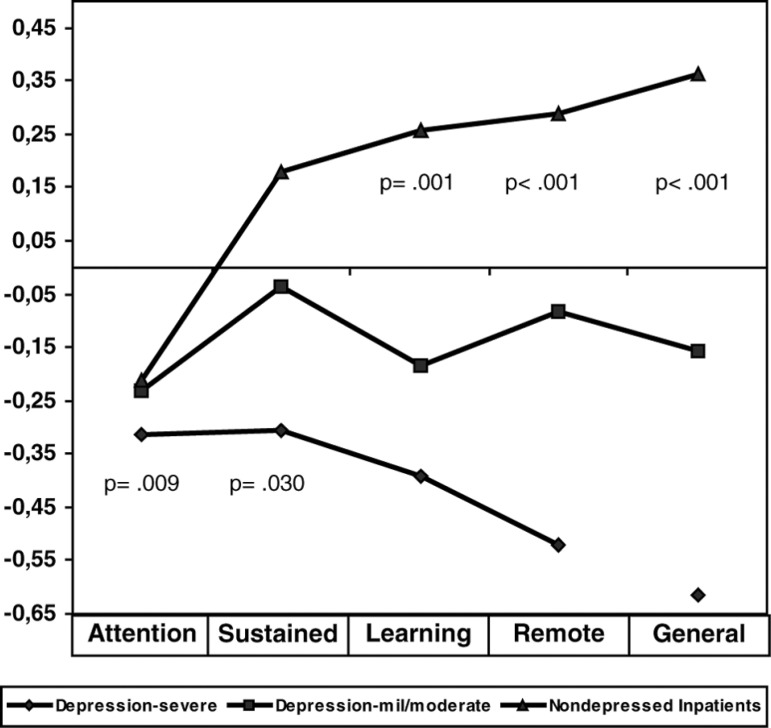
Domain z-scores for the three groups (severe depression, mild/moderate depression and medical inpatients).

The analysis of domains (sum of individual test z scores under definition) showed that attention, learning, remote memory, and general mental functioning were impaired in both severe and mild/moderate depressed patients compared to medical inpatients (age and education adjusted) (Figure 1). For the domain “sustained attention and working memory”, only severely depressed patients differed from the medical controls (p= 0.008). Severely depressed patients significantly differed from the mild/moderate on domains “Remote memory” and “General mental functioning” (p=0.024 and p= 0.016, respectively) (Figure 1). The severe patients presented the worst performances.

### INDEX (50% of positive tests) - logistic regression

For this model the independent variables age,Montgomery- Asberg depression scale, education, sex, and diagnostic status were used in the analysis.

With this index, 51% (N=23) of the severely depressed patients, 31% (N=13) of the mild/moderate depressed patients, and 19% (N=22) of the medical inpatients were identified (χ^2^=16.84; p=0.0001). Age (B= –0.042; p=0.006), Education (B=0.243; p=0.0001) and Diagnosis (B= –1.503; p=0.045) were the significant variables in the final model to explain the outcome. Age (OR=0.96), Education (OR=1.28) Diagnosis (OR=0.22) with 95% CI did not include the unit.

## Discussion

We aimed to evaluate performance on cognitive tests in a group of clinically depressed patients comparing with a group of cognitively normal medical inpatients, analyzing impact of education. Depression showed a significant effect upon cognition as well as education, but no interaction was observed between them. Age correlat-ed with almost all tests. However, Digit span, the immediate recall of Logical memory and Major Public Events did not present correlation with age in this sample. The main conclusion based upon these findings was the important association of depression, especially more severe forms of Major Depression, with general mental functioning, sustained attention and working memory, learning memory (verbal) and remote memory.

Several earlier studies have shown that patients with depression were impaired particularly on tests of verbal learning and memory^36^. Cognitive tasks may be sensitive to the effects of some antidepressants^37^ and most of our patients were under the effect of such medications. In our sample, we observed a significant effect of psychoactive drugs upon cognitive performance in general mental functioning, learning memory and sustained attention domains. However, a proportion (30%) of our patients was drug-free and was uniformly distributed between severe and mild/moderate groups, as were the other classes of drug use. We carried out analyses, controlled for drug effect, and cognitive/memory performances demonstrated the same independent effect from depression and education.

The effect of severity of depression was observed on five tests (corresponding to five different domains) in this sample. Although the effect of severity of depression on test performance has been measured in many studies by examining the correlation between depression rating scales, especially Hamilton´s, and test scores, the findings have been conflicting. Some studies reported no correlation between performance and severity of depression^38-42^, while others demonstrated this relationship^7,36,43-45^. Correlations could be sensitive to patient selection because the Hamilton Depression scale may be confounded by severe scores which are associated with more endogenous patterns of symptoms^16^. The Montgomery-Asberg Depression scale, on the other hand, covers ten depressive domains^28,29^. The Hamilton Depression Rating Scale (HAMD17)^46^ and the Montgomery-Åsberg Depression Rating Scale (MADRS)^28,29^ are the most extensively used observer instruments world-wide in clinical and psychopharmacological depression research to assess severity of depression after a categorical diagnosis has been ascertained^47^.

The MADRS is increasingly employed in clinical research because earlier studies had suggested the scale could be superior to the traditional HAMD_17_ with respect to sensitivity to change^30,48^ and other psychometric characteristics^49^.

Education has a significant influence on cognitive test performance. According to our findings, education can be an important confounder in establishing cognitive deficits related to depression. Groups with higher levels of education perform better on most neuropsychological tests^20-23^. On the other hand, low educational attainment may be responsible for false-positive responses in cognitive assessment. The impact of education associated to presence of diseases on cognitive tests or batteries has been extensively evaluated, even among subjects with lower attainment. There is extensive evidence that low education levels are linked to an indirect index of lower reserve capacity (i.e., a risk factor) which reduces the threshold for neuropsychological abnormality^50^.

Our study emphasized the independent effect of lower education and of diagnosis of depression. The applicability of neuropsychological tests and their performance in countries where rates of illiteracy and low socioeconomic levels are high, as is the case in Brazil, remains a very important issue to be debated. The sample was drawn from a city in which socioeconomic and educational characteristics are different from the majority of the other large Brazilian cities. This may suggest that similar investigations carried out in these locations could serve to demonstrate the practical problems of cross-cultural testing.

## Figures and Tables

**Table 5:** Final model of the logistic regression for cognitive impairment as the outcome.

Variables	B	SE	Wald	p value	OR	95% CI lower - upper	
Age	-0.042	0.015	7.610	0.006	0.959	0.931	0.988
Education	0.243	0.061	15.753	0.000	1.275	1.131	1.437
Diagnostic group (1)	-1.503	0.748	4.032	0.045	0.223	0.051	0.965
Montgomery-Asberg	-0.035	0.021	2.783	0.095	0.966	0.927	1.006
Sex (1)	-0.543	0.393	1.911	0.167	0.581	0.269	1.255
Constant	2.929	1.081	7.343	0.007	-	-	-

Learning/memory impairment defined as: at least 50% positive on the following tests coded as 0 impaired, 1 not impaired; Diagnostic Group coded 0 major depression, 1 medical inpatients; Sex coded 0 male, 1 female.
